# Tumour-derived Extracellular Vesicle and Particle Reprogramming of Interstitial Macrophages in the Lung Pre-Metastatic Niche Enhances Vascular Permeability and Metastatic Potential

**DOI:** 10.21203/rs.3.rs-4462139/v1

**Published:** 2024-05-30

**Authors:** Shani Dror, Serena Lucotti, Tetsuhiko Asao, Jianlong Li, Inbal Wortzel, Lee Shaashua Berger, Irina Matei, Nancy Boudreau, Haiying Zhang, David Jones, Jacqueline Bromberg, David Lyden

**Affiliations:** 1 Children’s Cancer and Blood Foundation Laboratories, Departments of Pediatrics, and Cell and Developmental Biology, Drukier Institute for Children’s Health, Meyer Cancer Center, Weill Cornell Medicine, New York, NY, USA; 2 Department of Thoracic Surgery, Memorial Sloan-Kettering Cancer Center, New York, NY 10065, USA; 3 Department of Medicine, Memorial Sloan Kettering Cancer Center, New York, NY, USA; 4 Department of Medicine, Weill Cornell Medicine, New York, NY, USA

## Abstract

Extracellular vesicles and particles (EVPs) are pivotal mediators of pre-metastatic niche formation and cancer progression, including induction of vascular permeability, which facilitates tumor cell extravasation and metastasis. However, the mechanisms through which EVPs exert this effect remain poorly understood. Here, we elucidate a novel mechanism by which tumor EVPs enhance endothelial cell permeability, tumor extravasation, and lung metastasis to different degrees, depending on tumor type. Strikingly, vascular leakiness is observed within 48h following tumor implantation and as early as one hour following intravenous injection of tumour-derived EVPs in naïve mice. Surprisingly, rather than acting directly on endothelial cells, EVPs first activate interstitial macrophages (IMs) leading to activation of JAK/STAT signaling and IL-6 secretion in IMs which subsequently promote endothelial permeability. Depletion of IMs significantly reduces tumour-derived EVP-dependent vascular leakiness and metastatic potential. Tumour EVPs that strongly induce vascular leakiness express high levels of ITGα5, and ITGα5 ablation impairs IM activation, cytokine secretion, and subsequently vascular permeability and metastasis. Importantly, IL-6 expression is elevated in IMs from non-involved tumor-adjacent lung tissue compared to distal lung tissue in lung cancer patients, highlight the clinical relevance of our discovery. Our findings identify a key role for IM activation as an initiating step in tumor type-specific EVP-driven vascular permeability and metastasis, offering promising targets for therapeutic intervention.

Metastasis is a critical phase of tumor progression, and it remains a primary challenge in treating cancer and a major cause of cancer mortality^1^. Therefore, elucidating the fundamental molecular and cellular constituents of each stage of metastatic development is critical to identify novel therapeutic targets. To establish metastasis, tumour cells must first intravasate into the circulation, survive in the blood circulation, then extravasate into distal tissues and colonize them^2^. A pivotal step in metastasis entails the extravasation of malignant cells from the bloodstream into distant tissues.

The vascular endothelial cell layers provide physical and immunological barriers to fluids, proteins, and cells^3^. In order to extravasate from the bloodstream to distant organs, cancer cells disrupt endothelial barrier integrity, leading to increased vascular permeability^3^. Primary tumours can facilitate metastasis through the secretion of soluble factors and extracellular vesicles and particles (EVPs), which create a supportive microenvironment in distal organ sites, enabling metastatic lesions to form. Our previous work demonstrated that creation of this pre-metastatic niche (PMN) precedes the arrival of cancer cells^4–7^. Vascular permeability is one of the hallmarks of lung PMN formation and represents an early step in the cascade leading to pulmonary metastasis^8–10^. Consequently, upon reaching the PMN, cancer cells exploit the increased vascular permeability to infiltrate the organ and establish metastatic lesions. Strategies aiming at reducing vascular endothelial permeability have shown promise in diminishing the metastatic burden^8,9,11^.

EVPs, which contribute to the formation of the PMN, are a diverse group of nanoscale vesicles actively released by cells^12^. EVPs selectively package cargo comprising proteins, RNA, DNA, and lipids along with their capacity for long-distance intercellular communication. Their importance is increasingly recognized in both normal physiological processes and pathological pathways, particularly in cancer development and metastasis^13,14^. Previous work from our group has demonstrated that within 24 hours post-administration of tumour EVPs, vascular leakiness is detected in both the lungs^6,15^ and brains^16^ of naïve animals. However, the mechanism by which these EVPs induce vascular permeability remains unexplored.

Previous studies have demonstrated that cancer cell-secreted soluble factors, including VEGF, TGF-β, and TNFα, are potent inducers of vascular permeability^17^. Elevation of Angpt2, MMP3, MMP10, and MMP9 during the PMN destabilized endothelial integrity as well^9,18^. In addition, EVPs can compromise the integrity of the endothelial barrier through multiple mechanisms. For instance, in breast cancer, brain cancer, and colorectal cancer, micro-RNAs encapsulated within EVPs have been shown to increase permeability by disrupting adherent and tight junctions, or via actin remodeling^11,19–21^. Other reports have also shown that EVPs can transfer proteins to endothelial cells, and these proteins can subsequently contribute to altering vascular permeability^22–24^. Moreover, induction of pro-apoptotic signals^25^ and necroptosis^26^ in endothelial cells led to vascular permeability and increase cancer cell extravasation. These findings underscore the importance of maintaining an intact endothelium as a defensive barrier against cancer cell extravasation. Nevertheless, the precise molecular mechanisms governing EVP regulation of vascular permeability in different cancers and throughout various phases of tumour progression are not well understood.

Here we set out to dissect the events that initiate vascular permeability by performing a comparative analysis of cancer models characterized by varying levels of vascular permeability in the lung. Surprisingly, we found that the influence of cancer cell-specific EVPs on vascular permeability was mediated through their effect on lung interstitial macrophages (IMs), rather than a direct effect on endothelial cells (ECs). Specifically, IMs which took up ITGα5- enriched EVPs, leading to the secretion of IL-6, which in turn induced vascular permeability in ECs. Furthermore, we demonstrated that lung-tropic EVPs induced permeability accompanied by increased incidence of extravasation and metastasis. Taken together these findings reveal a critical role for tissue resident macrophages in regulating vascular permeability, identifying IMs as a novel target for therapeutic modulation of vascular permeability.

## Results

### Tumour orthotopic models and tumour-derived EVPs induce varying degrees of vascular leakiness in the lung at early time points

Given the lung’s susceptibility to metastasis and that vascular leakiness is a hallmark of the lung PMN, we sought to identify tumour types capable of inducing pulmonary vascular permeability. To achieve this, we conducted a comparative analysis utilizing different tumour types known for their lung metastatic propensity in both mouse models and cancer patients. Specifically, we compared the following metastatic cell lines: B16F10 melanoma, K7M2 osteosarcoma, and 4T1 breast cancer. Additionally, the non-metastatic breast cancer cell line 67NR was used as a control.

**Figure 1:**
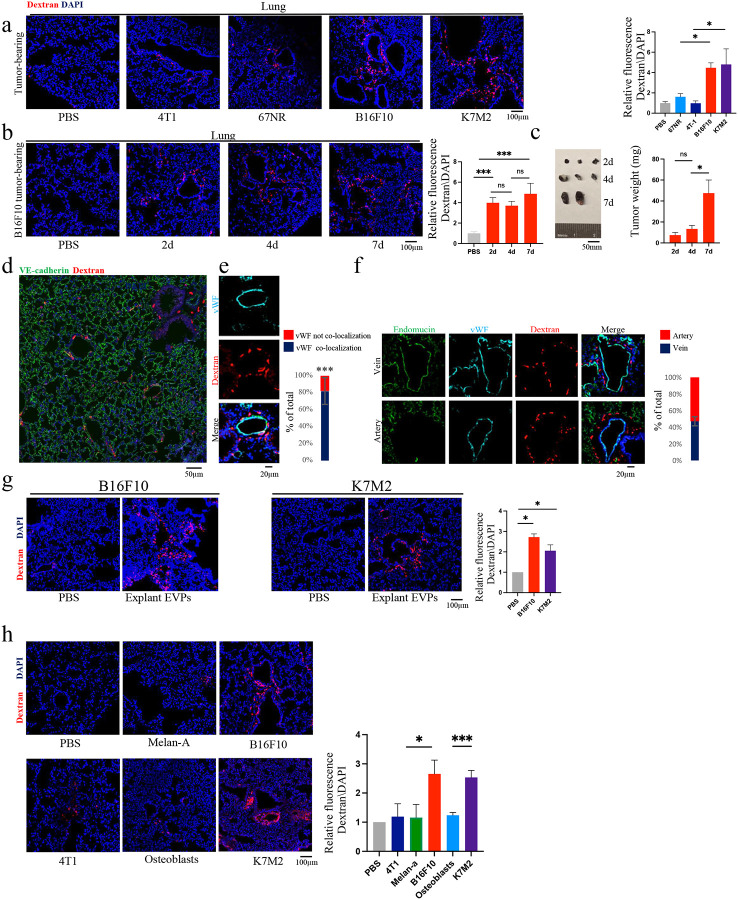
Tumour orthotopic models and tumour-derived EVPs induce various varying degrees of vascular leakiness in the lung **a-b**. Representative images (left) and associated statistical analysis(Right) of *in vivo* vascular permeability determined by the appearance of intravenously injected dextran (Red). DAPI-stained nuclei appear in blue (×20 Magnification). **a**. Lungs from 14-day tumour-bearing mice. Data represent the mean ± SEM (*n* = 3), p<0.05. Scale bar: 100 μm. **b**. Lungs from tumour-bearing mice at different time points. Data represent the mean ± SEM (*n* = 2), p<0.001. Scale bar: 100 μm. **c**. Representative images (left) and associated statistical analysis (right) of tumour weight (g) at Different time points. Data represent the mean ± SEM (*n* = 2). p<0.05. Scale bar: 50 mm. **d**. Immunofluorescence analysis (×20 Magnification). DAPI-stained nuclei appear in blue. Lung tissues exhibit expression of VE-Cadherin (green) and dextran (red). Scale bar: 50 μm. **e**. Immunofluorescence analysis (×40 Magnification) and associated statistical analysis. Left: DAPI-stained nuclei appear in blue. Lung tissues exhibit expression of vWF (cyan) and dextran (red). Right: quantification of the percentage number of dextran co-localized with surrounding vWF-positive blood vessels. Data represent the mean ± SEM (*n* = 5), p<0.05. Scale bar: 20 μm. **f**. Immunofluorescence analysis (×40 magnification) and associated statistical analysis. Left: DAPI-stained nuclei appear in blue. Lung tissues exhibit expression of Endomucin (green), vWF (cyan), and dextran (red). Right: Quantification of the percentage number of co-localized dextran with surrounding arteries or veins. Data represent the mean ± SEM (*n* = 3). Scale bar: 20 μm **g**. Representative images (left and middle) and associated statistical analysis (right) of *in vivo* vascular permeability determined by the appearance of intravenously injected dextran (red), 1 hour after administration of 10ug EVPs from B16F10 tumour (left) and K7M2 tumour (middle). DAPI-stained nuclei appear in blue (×20 magnification). Data represent the mean ± SEM (*n* = 2), p<0.05. Scale bar: 100 μm. **h**. Representative images (left and middle) and associated statistical analysis (right) of *in vivo* vascular permeability determined by the appearance of intravenously injected dextran (red), 1 hour after administration of 10ug EVPs from different cell lines. DAPI-stained nuclei appear in blue (×20 magnification). Data represent the mean ± SEM (*n* = 2), p<0.05. Scale bar: 100 μm.

To determine which of these models can induce vascular leakiness *in vivo*, we orthotopically implanted these cancer cell lines into mice and evaluated lung vascular permeability after 14 days, which is the timepoint when the lung PMN stage for these models. Notably, different tumour types induce varied levels of vascular leakiness in the lung. In particular, B16F10 and K7M2 tumours induced a significant degree (~4-fold increase) of lung vascular leakiness, compared to the breast cancer models (4T1, 67NR), which induced lower levels of vascular leakiness (Fig. 1a).

Subsequently, we aimed to identify the earliest time point at which changes in lung vascular permeability could be detected. B16F10 cells were orthotopically implanted into mice, and the extent of lung vascular leakiness was examined at the following intervals: 2 days, 4 days and 7 days following tumour implantation. We were able to detect lung vascular permeability as early as 2 days post tumour implantation. Notably, the increases in tumour weight at later time points did not result in further increases in vascular leakiness (Fig. 1b–c).

Our observations also suggested a potential correlation between the presence of dextran preferentially extravasated around larger blood vessels, rather than microvessels, within the lung. To investigate this phenomenon further, we stained lung tissue of mice bearing 14-day orthotopic tumours for von Willebrand Factor (vWF), which is strongly expressed in the endothelium of veins and arteries but not of capillaries^27^, along with VE-cadherin, which is homogeneously expressed in all endothelial cells in the lung^28^ (Fig. 1d and Extended Data Fig. 1a). Our findings revealed that more than 80% of leakiness was observed surrounding the larger vWF-expressing blood vessels, rather than in the capillaries (Fig. 1e). These results align with previous reports indicating that in the pulmonary circulation, the microvascular endothelium forms a considerably tighter barrier compared to arterial or venule endothelium^29,30^.

To investigate whether vascular permeability occurred in veins and/or arteries, we performed staining for endomucin, which is expressed in venous and capillary endothelium but not in most arterial endothelium^31,32^. We observed that dextran leakiness was evident to a similar extent around both veins and arteries in the lungs of B16F10 tumour-bearing mice 14 days following implantation (Fig. 1f). This finding indicates that macro-vessels are susceptible to tumourinduced vascular leakiness at very early stages of cancer progression, emphasizing the fundamental role of leakiness in this process during the initial stages of metastasis progression.

We previously showed that vascular leakiness can be observed in the lung 24 hours after melanoma-derived EVP administration^15^. However, considering that vascular leakiness becomes evident as early as 48 hours following tumour implantation, our objective was to identify the earliest time point at which leakiness could be observed upon tumour EVP administration. Remarkably, we discovered that there wasn’t a significant difference in the degree of vascular permeability observed one-hour versus 24-hours following retro-orbital injection of 10 μg of B16F10 EVPs (Extended Fig. 1b). As the one-hour time interval would enable us to investigate the specific effect of EVP-induced vascular leakiness separately from other potential effects of EVPs, we focused on the one-hour time point for further investigation.

Next, to validate whether tumour EVPs could induce vascular permeability to a degree similar to that observed in tumour-bearing mice, we isolated EVPs from B16F10 and K7M2 tumour-derived explants and administered them by retro-orbital injection into naïve mice. Remarkably, a single injection of both B16F10 and K7M2 tumour EVPs led to significant leakiness (~2.7 and ~2 fold increase) compared to control within one hour after EVP administration which was similar to that observed in tumour-bearing mice (Fig. 1g).

To confirm that vascular leakiness was induced by tumour cell-derived EVPs and not by stromal cell-derived EVPs within the tumour microenvironment, we isolated EVPs from B16F10 and K7M2 cancer cell lines. Following retro-orbital administration of these EVPs into mice, we assessed their uptake and downstream effects on the lung 1 hour post injection. While all EVPs were taken up by the lung to varying degrees (Extended Fig 1c), not all induced vascular permeability to a significant extent. Consistent with our findings in tumour-bearing mice, EVPs derived from B16F10 and K7M2 caused a significantly higher level of vascular leakiness, as compared to EVPs from 4T1, Melan-A (normal melanocyte cell line), and primary osteoblasts which did not induce a significant response (Fig. 1h). Furthermore, to rule out any potential contribution of non-EVP soluble factors, we retro-orbitally administered EVP-depleted conditioned media (CM) from B16F10 or K7M2 cells into mice. The CM was concentrated from the same volume required to isolate 10 μg of EVPs. In this case, we did not observe an increase in lung permeability when we injected CM alone (Extended Fig. 1d), indicating EVPs, and not other soluble factors, are necessary to induce vascular leakiness.

### Acute vascular leakiness promotes cancer cell extravasation and lung metastasis

**Figure 2:**
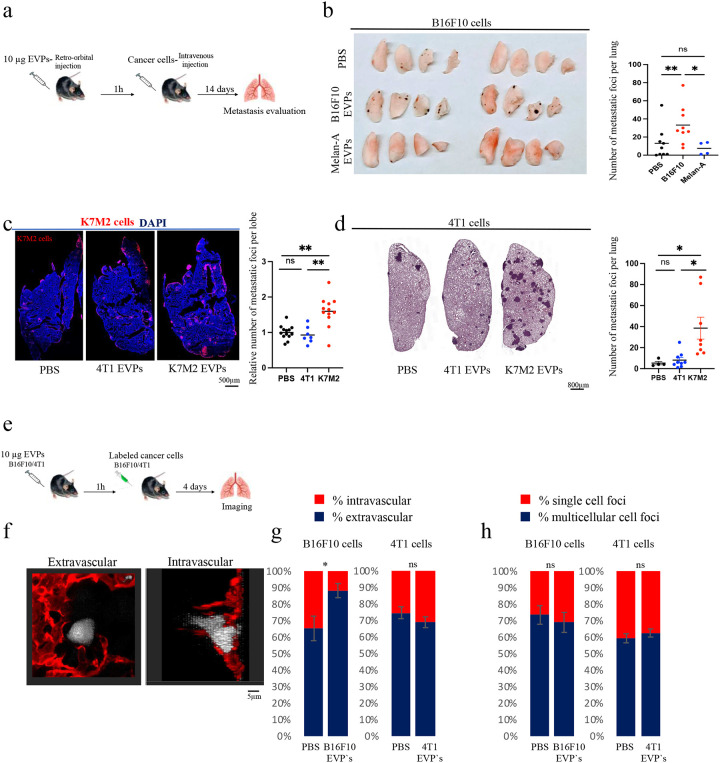
Acute leakiness promotes cancer cell extravasation and lung metastasis **a**. Schematic illustration of the experiment. **b**. Representative images of mice’s lungs at two weeks after tail vein injection with 50,000 B16F10 cells (left) and associated statistical (right) of relative number of macro-mets compared to PBS. Data represent the mean ± SEM (*n* = 3), p<0.05. **c**. Representative immunofluorescence imaging of lung’s lobe at two weeks after tail vein injection with 300,000 K7M2 cells and associated statistical analysis of relative metastasis number compared to PBS. DAPI-stained nuclei appear in blue and K7M2 cells in red. Data represent the mean ± SEM (*n* = 3), p<0.01. Scale bar: 500 μm. **d**. Representative H&E staining of lungs of mice after tail vein injection with 50,000 4T-1 cells for 14 days and associated statistical analysis for number of metastases. Data represent the mean ± SEM. (*n* = 3) p<0.05. **e**. Schematic illustration of extravasation experiment. **f**. Representative immunofluorescence imaging of mouse lung tissue 4 days post-tail vein injection with labeled B16F10 cells. Lung tissues exhibit expression of CD31 (red) and B16F10 cells (white). Scale bar: 5 μm. **g**. Quantification of the percentage of B16F10 cells (left) or 4T1 cells (right) extravasating into the tissue relative to the total cell count. Data represent the mean ± SEM (*≈*3), p<0.05. **h**. Quantification of the percentage of single-cell foci or multicellular g extravasation into the tissue, relative to the total cell count. Data represent the mean ± SEM (*n* = 3).

Having observed significant changes in vascular permeability within one hour following tumour cell-derived EVP injection, we sought to investigate whether this was sufficient to promote cancer cell extravasation and metastatic outcome in the lung. To explore this, we injected EVPs from tumours that could induce varying degrees of leakiness, followed by tail vein injection of the corresponding tumour cells after one hour, and macroscopic and microscopic metastasis assessment 14 days later (Fig. 2a). Specifically, we compared the effects of B16F10 EVPs (high leakiness) with normal Melan-A EVPs (no leakiness), as well as the effects of K7M2 EVPs (high leakiness) with 4T1 EVPs (low leakiness) (Fig. 2). The injection of B16F10 EVPs one hour prior to B16F10 cell injection caused a 2.5-fold increase in metastatic foci compared to the PBS control group (Fig. 2b–c). Conversely, the administration of Melan-A EVPs, one hour prior to B16F10 cell injection, did not affect the number of metastatic foci compared to the PBS control (Fig. 2b). Similarly, the injection of K7M2 EVP one hour preceding K7M2 cell injection led to an approximately 1.6-fold significant increase in the number of metastatic foci compared to the PBS treatment group. In contrast, administration of 4T1 EVPs one hour before injection of 4T1 cells did not increase lung metastases (Fig. 2c).

Furthermore, to determine if tumour EVP-mediated leakiness could potentiate metastatic seeding of cancers that inherently are not inducing leakiness themselves, we investigated the effects of administering K7M2 EVPs one hour before the injection of 4T1 cells. Fourteen days later, we observed a significant (~7-fold) increase in the number of 4T1 lung metastases in mice pre-treated with K7M2 EVPs, compared to the number of 4T1 metastases in mice pre-treated with PBS treatment (Fig. 2d). Notably, the administration of 4T1 EVPs one hour prior to injection of 4T1 cells did not increase 4T1 lung metastases (Fig. 2d). Together, these results highlight that an increase in tumour EVP-mediated vascular permeability can potentiate the tumour cell intrinsic metastatic potential even in the case of highly metastatic cells such as 4T1, which induce limited vascular leakiness themselves.

To confirm that the increase in metastases was primarily linked to EVP-induced vascular leakiness rather than other secondary effects caused by EVPs, we administered histamine retro-orbitally, a well-known inducer of vascular permeability in the lung^33^, as a positive control (Extended Fig. 2a). Histamine alone induced vascular permeability and elevated the number of metastases to a similar degree as B16F10-derived EVPs (Extended Fig. 2b–c) following 14 days. However, when we administered EVPs 1 hour prior to histamine, there was no further increase in the number of metastases compared to histamine or EVPs alone (Extended Fig. 2b–c). This finding indicates that the increased metastasis upon EVP treatment can be attributed primarily to the altered endothelial barrier integrity, and not to other potential effects of EVP cargo. This supports the notion that vascular permeability represents one of the first pro-metastatic processes triggered by EVPs.

Prior research showed that following tail vein injection, the majority of B16F10 cells extravasate from the vasculature into the lungs by day 4, and the cells failing to do so most likely die^34,35^.To further investigate if the increase in metastases is related to the ability of cells to extravasate, we labeled tumour cells with a cytoplasmic dye prior to tail vein injection. To visualize lung endothelium, mice were injected retro-orbitally with CD31-PE antibody, followed by lung perfusion and whole organ 3D lung imaging^34^. We then analyzed the percentage of tumour cells extravasated into the lung parenchyma and the proportion of multicellular tumour cell foci by day 4 post intravenous injection (Fig. 2e, f, and Extended Fig. 2c). Remarkably, we observed that a single dose of B16F10 EVPs one hour prior to cancer cell injection was sufficient to significantly increase (~35%) B16F10 cell extravasation into the lungs (Fig. 2g). Conversely when mice were injected with 4T1 EVPs which did not induce vascular leakiness, we did not detect any increase in cell extravasation of the cells into the lung parenchyma was detected (Fig. 2g). Moreover, we did not detect any difference in the percentage of multicellular cell foci of cells that had extravasated by day 4 between the PBS control group and the EVPs treated group in either model (Fig. 2h). These findings indicate that vascular permeability, rather than enhanced proliferation, predominantly contributes to the process of metastatic seeding of the lungs.

### Lung vascular leakiness is mediated by interstitial macrophages

**Figure 3:**
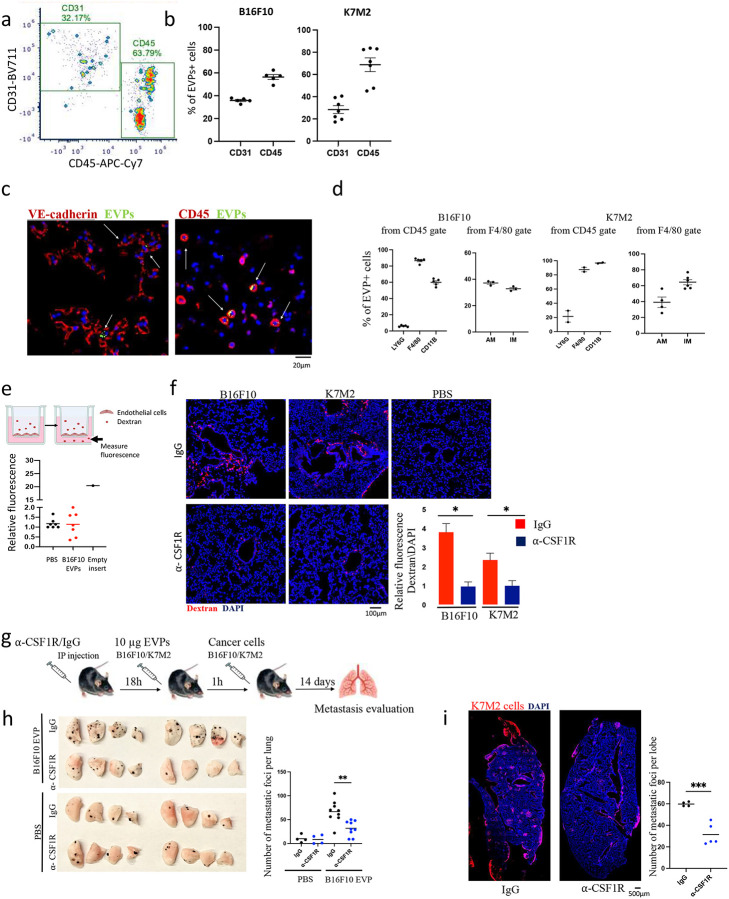
Lung vascular leakiness is mediated by interstitial macrophages **a-d**. B16F10 and K7M2-derived EVPs were labeled with CellVue Burgundy, PBS, and dye only serves as control. The EVPs (10 μg) were injected retro-orbitally, and 1h later, the lungs were extracted and analyzed by flow cytometry. Data represent the mean ± SEM (*n* = 3). **a**. Representative image of FACS analysis of EVPs+ cells from total live cells. **b**. FACS analysis of the percentages of EVP+CD31+ endothelial cells and EVP+CD45+ immune cells from total EVPs+ cells. **c**. Representative image of immunofluorescence analysis (×40 magnification). DAPI-stained nuclei appear in blue. Lung tissues exhibit expression of VE-cadherin (left) and CD45 (right) with labeled EVPs (green). Scale bar: 20 μm. **d**. FACS analysis of percentages of EVP+ cells in different immune populations. **e**. Top: Schematic illustration of *in vitro* permeability experiment. Bottom: Effect of B16F10/PBS EVPs, on the permeability of HPAEC monolayers by an *in vitro* permeability assay. Data represent the mean ± SEM (*n* = 3). **f**. Representative images (left) and associated statistical analysis (right) of *in vivo* vascular permeability determined by the appearance of intravenously injected dextran (red). DAPI-stained nuclei appear in blue (×20 magnification). Data represent the mean ± SEM (*n* = 3), p<0.05. Scale bar: 100 μm. **g**. Schematic illustration of education experiment with IMs depletion. **h**. Representative imaging of mice lungs at two weeks after tail vein injection with B16F10 cells (left) and associated statistical analysis (right). Data represent the mean ± SEM (*n* = 3), p<0.01. **i**. Representative immunofluorescence imaging of mouse lung tissue following two weeks post tail vein injection with K7M2 cells and associated statistical analysis for metastasis number. DAPI-stained nuclei appear in blue and K7M2 cells in red. Data represent the mean ± SEM (*n* = 3), p<0.001 Scale bar: 500 μm.

To understand how EVPs increase vascular permeability, we next sought to determine which cell populations took up tumour-derived EVPs. EVPs derived from either B16F10 or K7M2 cells were labeled with lipophilic dyes and administered retro-orbitally to C57BL/6 and Balb/c mice, respectively, followed by lung flow cytometry analysis one-hour post injection. We found that CD31^+^ endothelial cells accounted for approximately 30%−40% of all EVP positive cells in the lung, while CD45^+^ immune cells contributed to 60%−70% of the uptake in both models (Fig. 3a,b and Extended Fig. 3a). This observation was further validated through immunofluorescence staining (Fig. 3c). Since the majority of EVPs were taken up by immune cells, we further characterized immune populations taking up EVPs in the lung (Fig. 3d). We found that F4/80+ macrophages were responsible for ~80% of the total CD45^+^ EVP uptake. Within the macrophage subsets, CD11B+,F4/80+,Siglec-F-, LY6C- interstitial macrophages (IMs)^36^, accounted for ~40% and ~70% of the internalized B16F10 and K7M2 EVPs respectively, while CD11B+,F4/80+,Siglec-F+ alveolar macrophages (AMs)^36^ accounted for the remaining EVPs. Additionally, in B16F10 EVP and K7M2 models, approximately 7% and 20% of CD45^+^ cells, respectively, were neutrophils (CD11B+, LY6G+)^36^ (Fig. 3d).

As endothelial and immune cells constituted the primary populations taking EVPs in the lung, we aimed to identify the specific cell type responsible for disrupting the endothelial barrier. To this end, we performed an *in vitro* permeability assay, quantifying the fluorescence intensity of rhodamine-labeled dextran that passed through monolayers of lung primary pulmonary artery endothelial cells (HPAEC) cultured on 3-mm transwell inserts. Surprisingly, the direct administration of B16F10 EVPs to lung endothelial cells did not yield any discernible alterations in permeability (Fig. 3e).

Next, we asked whether lung resident macrophages were functionally required for tumour EVP-induced vascular permeability. Thus, we depleted AMs using clodronate liposomes, IMs using anti-CSF1R, and neutrophils by anti-Ly6G antibodies. We confirmed depletion specificity and efficiency for each treatment (Extended Fig. 3c). Importantly, AM or neutrophil depletion did not result in any noticeable alterations in permeability following EVP administration (Extended Fig. 3d). However, when IMs were depleted, a significant (>80% and >55%) reduction in leakiness was observed following B16F10 and K7M2 EVP administration, respectively (Fig. 3f).

Finally, to determine the requirement for IM-dependent vascular leakiness for lung metastasis, we evaluated EVP-induced metastasis in the lung following IM depletion. Specifically, we administered a single intraperitoneal injection of either anti-CSF1R or IgG antibodies 18 hours prior to EVP treatment. One hour following the administration of EVP, mice were intravenously injected with cancer cells (Fig. 3g). We observed that depletion of IMs significantly abrogated the pro-metastatic effect of EVP treatment and reduced the number of metastatic lesions in the lungs by ~50% for both melanoma and osteosarcoma models (Fig. 3h, i). Together, these data indicate that IMs are essential for EVP-dependent vascular leakiness and metastasis promotion.

### IL-6 secretion by interstitial macrophages enhances vascular permeability

**Figure 4:**
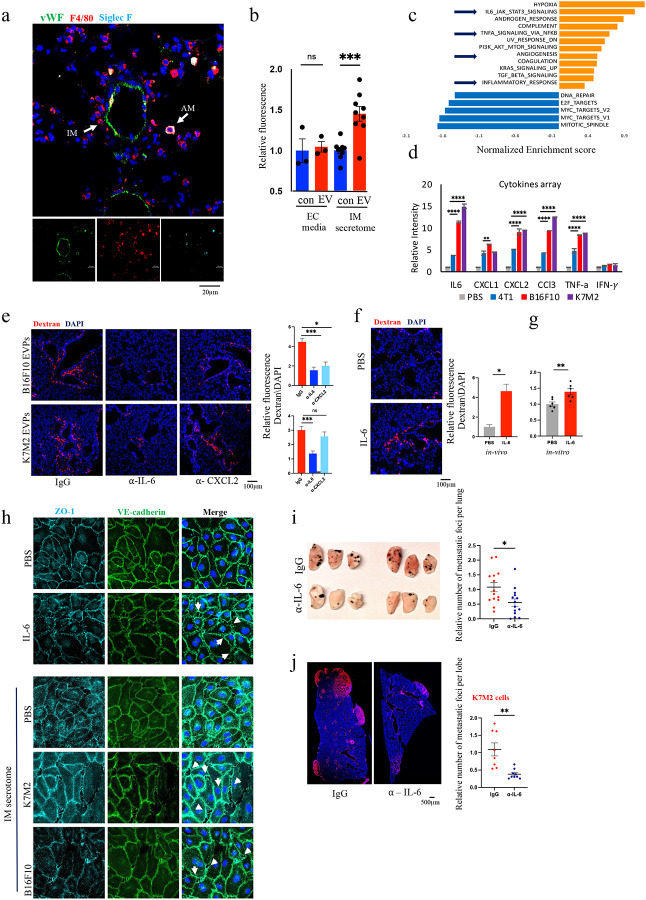
IL-6 secretion by interstitial macrophages enhances vascular permeability **a**. Representative lung imaging of immunofluorescence analysis (×40 magnification). DAPI-stained nuclei appear in blue. Lung tissues exhibit expression of F4/80 (red), Siglec-F (cyan), and vWF (green). Arrow indicate representive AM and IM cell. Scale bar: 20 μm. **b**. The effect of B16F10-treated IM secretome on the permeability of HPAEC monolayers assessed by an *in vitro* permeability assay. Data represent the mean ± SEM (*n* = 3). **c**. GSEA of the common differentially expressed genes using Hallmark gene sets, showing significantly changed signaling pathways with false discovery rate (FDR) < 0.1. Gene lists for signaling pathways are shown in Supplementary Table 2. NES, normalized enrichment score. **d**. Analysis of cytokine array of the secretome from IMs 3h after treatment with B16F10/K7M2/4T-1 EVPs or PBS. **e**. Representative images (left) and associated statistical analysis (right) of *in vivo* vascular permeability determined by the appearance of intravenously injected dextran (red). DAPI-stained nuclei appear in blue (×20 magnification). Data represent the mean ± SEM (*n* = 3), p<0.05. Scale bar: 100 μm. **f**. Representative images (left) and associated statistical analysis (right) of *in vivo* vascular permeability following IP injection of 500nM IL-6 protein. DAPI-stained nuclei appear in blue. (×20 Magnification). Data represent the mean ± SEM (*n* = 2), p<0.05. Scale bar: 100 μm. **g**. Effect of IL-6 (40nM) recombinant protein on the permeability of HPAEC monolayers by *in vitro* permeability assay. Data represent the mean ± SEM (*n* = 2). **h**. Immunofluorescence analysis (×40 Magnification). DAPI-stained nuclei appear in blue. HAPEC exhibits expression of ZO-1(cyan) and VE-Cadherin (green). HAPEC was treated with 40nM IL-6 (left) and IMs` secretome (right) for 1h. Scale bar: 20 μm. **i**. Representative imaging of mice’s lungs at two weeks after tail vein injection with 50,000 B16F10 cells (left) and associated statistical analysis for number of metastasis (right). Data represent the mean ± SEM (*n* = 3), p<0.01. **j**. Representative immunofluorescence imaging of lung’s lobe at two weeks after tail vein injection with 300,000 K7M2 cells (left) and associated statistical analysis for relative number of metastasis (right). DAPI-stained nuclei appear in blue and K7M2 cells in red. Data represent the mean ± SEM (*n* = 2), p<0.001. Scale bar: 500 μm.

As shown in Fig. 1e, EVP-dependent vascular leakiness occurred in large vWF+ blood vessels in the lung. IMs are primarily situated in the interstitial space between the microvascular endothelium and alveolar epithelium, suggesting paracrine signaling between EVP+ IMs and adjacent endothelial cells (Fig. 4a)^37^. Notably, immunofluorescence analysis revealed that, in contrast to F4/80+, Siglec-F+ AMs^36^, F4/80+, Siglec-F- IMs^36^ are in close proximity to vWF+ cells.

To explore the potential role of cytokine secretion by IMs in mediating vascular permeability, we isolated IMs from murine lung tissue and exposed them to B16F10 EVPs (1μg/ml, 3 hours). We then isolated conditioned media (CM) from untreated control or B16F10 EVP-treated IMs and applied the CM onto HPAEC cells in an *in vitro* permeability assay. Remarkably, we observed a significant (45%) increase in dextran permeability when exposed to the EVP-treated IM secretome as compared to the untreated IM secretome (Fig. 4b). Likewise, a significant (30%) increase in HPAEC permeability was observed when endothelial cells were exposed to the IM secretome derived from K7M2 EVP treated IMs, as opposed to 4T1 EVP treated IMs (Extended Fig. 4a). This highlights the tumour specificity of EVP-dependent reprogramming of IMs.

To identify the functional changes in IMs following uptake of EVPs, we performed RNA sequencing (RNA-seq) of sorted IMs which had taken up fluorescently labeled B16F10 EVPs *in vivo* (Supplementary Table 1). Gene set enrichment analysis (GSEA) revealed that the IL-6-STAT3 signaling pathway, inflammation, TNFα signaling, and angiogenesis-related pathways were all significantly induced by B16F10 tumour cell-derived EVPs (Fig. 4c, Extended Fig. 4b, and Supplementary Table 2). As macrophage responses most often includes cytokine secretion, we set to identify the specific cytokines induced in IMs by the uptake of EVPs, by conducting a cytokine array assay and direct RNA expression. Compared to the IMs treated with 4T1 EVPs, IMs treated with B16F10 or K7M2 EVPs significantly enhanced the secretion of IL-6, CXCL2, CCL3, and TNF-α (Fig. 4d and Extended Fig. 4c). In agreement with the cytokine array, treatment of IMs with EVPs that do not induce leakiness (4T1 and Melan-A) did not alter IL-6, CXCL2, CCL3, and TNF-α cytokine mRNA expression, while treatment with B16F10 and K7M2 leakiness-inducing EVPs, significantly increased the expression of IL-6, CXCL2, CCL3, and TNF-α in IMs (Extended Fig. 4d).

To dissect the individual contributions of these cytokines to vascular leakiness, we treated mice with neutralizing antibodies against IL-6, CXCL2, CCL3, and TNF-α 18 hours prior to administering B16F10 or K7M2 EVPs, followed by dextran one hour later. Blocking CCL3 or TNF-α did not yield noticeable differences in lung vascular permeability in either model (Extended Fig. 4e). Neutralizing CXCL2 reduced lung vascular permeability in B16F10 EVP-treated mice but not in K7M2-treated mice. Importantly, neutralizing IL-6 prior to EVP administration significantly reduced lung vascular permeability in mice treated with either B16F10 or K7M2 EVPs (Fig. 4e).

The ability of IL-6 to induce vascular permeability is well-established both *in vivo* and *in vitro*^38,39^. Indeed, treatment with recombinant IL-6 enhanced vascular permeability both *in vivo* and in the *in vitro* HPAEC model (Fig. 4f, g). Immunofluorescence imaging of endothelial cells treated directly with recombinant IL-6 (40 nM) or exposed for 60 minutes to CM derived from IMs stimulated by B16F10 and K7M2 EVPs yielded similar findings. We observed alterations in VE-cadherin and ZO-1, consistent with endothelial barrier integrity disruption (Fig. 4h). Importantly, in mice depleted of IMs, administration of IL-6 alone was sufficient to induce significant vascular leakiness, suggesting it acts downstream of IM activation and upstream of EC dysfunction, and that it is sufficient to compensate for the effects of IM activation by tumour EVPs (Extended Fig. 4f), further suggesting IL-6-mediated signaling between IMs and endothelial cells.

Furthermore, when administering B16F10 or K7M2 EVPs in mice pre-treated with neutralizing antibodies against IL-6, administration of B16F10 or K7M2 EVPs led to a significant decrease in number of metastatic lesions over 14 days compared to isotype control antibody (Fig. 4i,j).

Together, these results show that EVP-induced secretion of IL-6 by lung IMs is required for vascular leakiness to support extravasation of circulating cancer cells and metastasis formation.

### Integrin-α5 in EVPs induces vascular leakiness and metastasis

**Figure 5:**
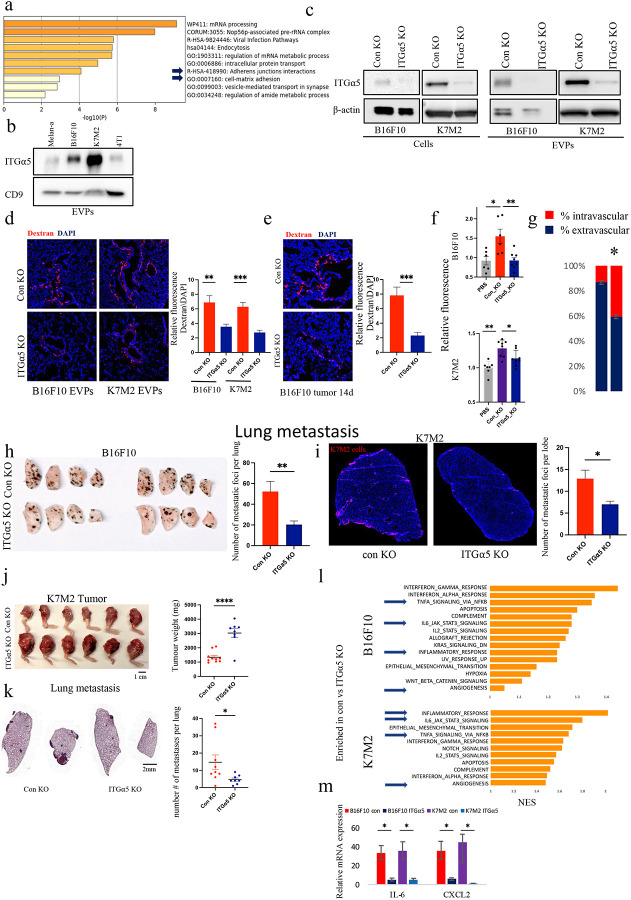
Integrin-α5 in EVPs induces vascular leakiness and metastasis **a**. Pathway analysis of proteins shared between B16F10 and K7M2 EVPs and absent in melan-A and 4T1 EVPs. **b**. Western blot analysis of ITGα5 in EVPs from different cancer cell lines. CD9 was used as a loading control. **c**. Western blot analysis of ITGα5 expression in B16F10 and K7M2 EVPs infected with vector control (Con KO) or ITGα5 KO virus. **d-e**. Representative images (left) and associated statistical analysis (right) of *in vivo* vascular permeability. DAPI-stained nuclei appear in blue (×20 magnification). Data represent the mean ± SEM (*n* = 3), p<0.01. Scale bar: 100 μm. **f**. Effect of CON KO and ITGα5 KO EVPs on the permeability of HPAEC monolayers by an *in vitro* permeability assay. Data represent the mean ± SEM (*n* = 2), p<0.01. **g**. Quantification of the percentage of B16F10 cells extravasating into the tissue relative to the total cell count, following education with CON KO and ITGα5 KO EVPs. Data represent the mean ± SEM (*n* = 3), p<0.05. **h**. Representative imaging of mice’s lungs at two weeks after tail vein injection with 50,000 B16F10 cells (left) and associated statistical analysis (right). Data represent the mean ± SEM (*n* = 3), p<0.01. **i**. Representative immunofluorescence imaging of lung’s lobe at two weeks after tail vein injection with 300,000 K7M2 cells. DAPI-stained nuclei appear in blue and K7M2 cells in red and associated statistical analysis for relative number of metastasis. Data represent the mean ± SEM (*n* = 2), p<0.05. Scale bar: 500 μm. **j**. Representative imaging of mice’s tumours at 4 weeks after intratibial injection with 1*10^6^ K7M2 cells (left) and associated statistical analysis (right). Data represent the mean ± SEM (*n* = 1), p<0.001. Scale bar: 1 cm. **k**. Representative H&E imaging of mice’s lungs at 4 weeks after intratibial injection with 1*10^6^ K7M2 cells (left) and associated statistical analysis (right). Data represent the mean ± SEM (*n* = 1), p<0.05. Scale bar: 1 mm. **l**. GSEA of the common differentially expressed genes using Hallmark gene sets, showing significantly changed signaling pathways with false discovery rate (FDR) < 0.1. Gene lists for signaling pathways are shown in Supplementary Table 7. NES, normalized enrichment score. **m**. RT-PCR analysis from IMs treated with CON KO or ITGα5 KO EVPs from B16F10 and K7M2 cells for 3h.

Although EVPs derived from the different cancer cell lines were all taken up in the lung (Extended Fig. 1f), only EVPs derived from a subset of cell lines induced robust vascular leakiness (Fig. 1i). To identify the cargos within EVPs contributing to endothelial permeability, we used mass spectrometry to compare EVP proteins from B16F10, K7M2, 4T1, and Melan-A (Supplementary Table 3). Pathway analysis of proteins shared between B16F10 and K7M2 EVPs (associated with high vascular leakiness) and absent in Melan-A and 4T1 EVPs (associated with low vascular leakiness), revealed significant enrichment of proteins involved in the “adherent junction interaction” and “cell-matrix adhesion” pathways (Fig. 5a and Supplementary Table 4). Cell adhesion molecules are known to play a pivotal role in mediating metastasis and extravasation and we previously showed that integrins on EVPs are implicated in cell-cell communication, organotropism, and cancer progression^6,40^. Therefore, to determine which EVP protein(s) are involved in vascular permeability, we first compared the expression of known adhesion proteins from the GSEA adhesion gene set with the adhesion proteins identified in our mass spectrometry results (Supplementary Table 5). We observed that a select few proteins, including Cadherin-2 (CDH2), Neural cell adhesion molecule 1 (NCAM1), and integrin-α5 (ITGα5) exhibited high expression in B16F10 and K7M2 EVPs, but were low or absent in the control EVP groups (Extended Fig. 5a). Levels of these proteins in EVPs were validated by Western blot analysis (Fig. 5b and Extended Fig. 5b).

To evaluate the functional impact of these proteins on vascular permeability in the lung, we used knockdown and knockout methods to reduce the expression of CDH2, NCAM1, and ITGα5 in B16F10 and K7M2 cell lines. Reduced expression was confirmed via Western blot analysis (Fig. 5c and Extended Fig. 5c). Notably, while knockdown of CDH2 and NCAM1 did not alter vascular leakiness (Extended Fig. 5d), a significant (~50%) reduction in the lung vascular leakiness was observed in mice treated with ITGα5 KO B16F10 or K7M2 EVPs relative to controls (Fig. 5d).

Next, we verified that reduced permeability was not due to changes in the production, morphology, or uptake of the EVPs in the lung. The loss of ITGα5 did not affect vesicle morphology, as examined by transmission electron microscopy (Extended Fig. 5e) or size distribution, as analyzed by nanoparticle tracking analysis (Extended Fig. 5f). There was a slight increase in the number of secreted K7M2 ITGα5 KO-derived EVPs, but not in the B16F10 model (Extended Fig. 5f). Importantly, loss of ITGα5 from EVPs did not interfere with the uptake of EVPs in general or the cell type specific uptake by either CD45^+^ and CD31^+^ cells. Specifically, there was no difference in the uptake of ITGα5 KO EVPs by IMs (Extended Fig. 5g, i). Thus, ITGα5 loss does not impact EVP formation or uptake by IMs.

We next orthotopically implanted B16F10 wildtype or ITGα5 KO cells into mice and analyzed vascular leakiness in the lung PMN of tumour-bearing mice 14 days after tumour implantation. ITGα5 loss didn’t affect primary tumor growth (Extended Fig 5j), however, lung vascular permeability was significantly decreased by ~70% in mice bearing ITGα5 KO orthotopic tumours (Fig. 5e). Similarly, the ability of EVPs derived from B16F10 and K7M2 cells lacking ITGα5, to induce permeability in *in vitro* assays was significantly reduced (Fig. 5f). The reduced permeability was accompanied by a significant reduction (~30%) in B16F10 cells extravasating to the lung in mice injected with a single dose of B16F10 ITGα5 KO EVPs, as compared to control KO EVPs (Fig. 5g).

Subsequently, we determined the metastatic potential of wild-type B16F10 or K7M2 cells injected into the tail vein one hour after a single dose of control and KO EVPs. Notably, in both models, mice treated with ITGα5 KO EVPs developed significantly (~50%) fewer metastases (Fig. 5h–i). To further determine whether ITGα5 can promote metastasis in orthoptic tumour model, we performed an education experiment. BALB/c mice were treated with control KO or ITGα5 KO K7M2-derived EVP every other day for three weeks. WT K7M2 tumor cells were implanted intratibially and allowed to grow for 4 weeks. We found that the tumour weight in mice educated with ITGα5 KO EVPs was significantly higher (~2.3 fold) compared to mice educated with control KO EVP (Fig. 5j). Conversely, mice treated with ITGα5 KO EVP exhibited a significantly lower incidence of metastasis (~2.5 fold) compared those treated with control EVPs (Fig. 5k). These data suggest that ITGα5-enriched tumour EVPs are crucial for inducing lung vascular permeability, facilitating tumour cell extravasation, and thus promoting metastasis.

Finally, to demonstrate the influence of ITGα5 in IM activation-dependent vascular permeability, we conducted RNA sequencing of isolated lung IMs treated for 3 hours with ITGα5 KO or control KO EVPs from both B16F10 and K7M2 cell lines (Supplementary Table 6). GSEA yielded consistent findings in both models: IMs treated with ITGα5 KO EVPs failed to upregulate IL-6-STAT3 signaling and inflammation pathways compared to cells treated with control EVPs (Fig. 5l, Extended Fig. 5k, l and Supplementary Table 7). The IL-6-STAT3 signaling pathway identified by RNA-seq was further confirmed by RT-PCR, which revealed that mRNA levels of IL-6 and CXCL2 were significantly lower in IM cells treated with ITGα5 KO EVPs compared to controls (Fig. 5m). Together, these data show the ITGα5 in EVPs is essential for activation of IL-6-STAT3 signaling, and induction of vascular leakiness.

**Figure 6:**
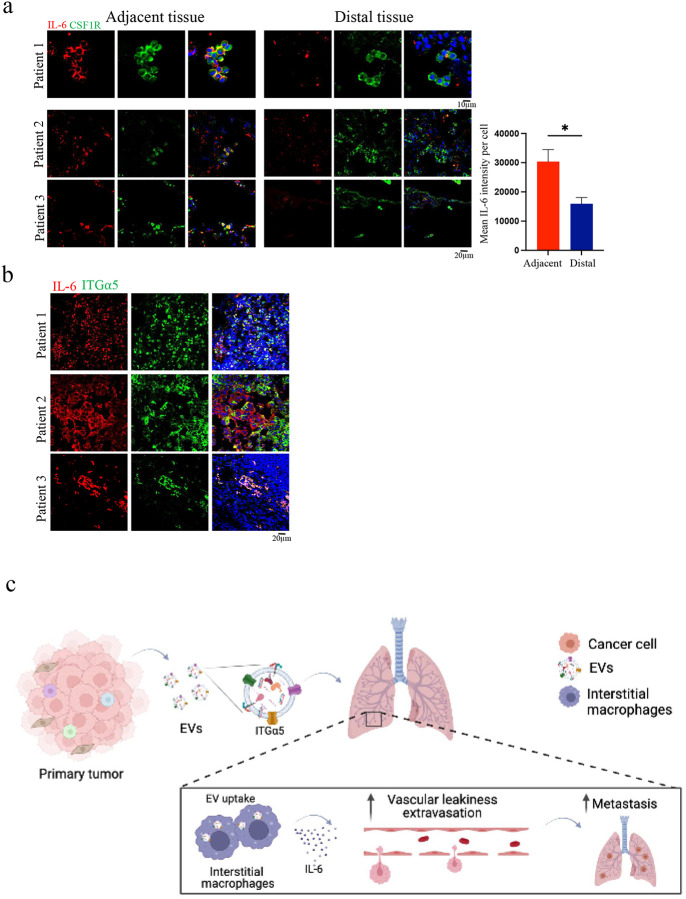
IL-6 expression is elevated in IMs within tumour-adjacent tissues compared to distant tissues. **a**. Representative images from 3 different patients (left) and associated statistical analysis of 8 different patients (right). (×40 Magnification). DAPI-stained nuclei appear in blue. Human lungs exhibit expression of IL-6 (red) and CSF1R (green) (n=8), Scale bar: 10/20 μm. **b**. Immunofluorescence Analysis (×40 Magnification). DAPI-stained nuclei appear in blue. Human lung cancer tumours exhibit expression of IL-6 (red) and ITGα5 (green). Scale bar: 20 μm. **c**. Model for EVP-mediated vascular permeability in the lung. Tumour-derived EVPs expressing ITGα5 target interstitial macrophages in the lung. This interaction stimulates the secretion of the cytokine IL-6, which, in turn, enhances the permeability of endothelial cells. This increased permeability facilitates the extravasation of cancer cells and ultimately promotes metastasis.

Moreover, analysis of lung tissues from patients diagnosed with lung cancer (Stages IAI-IIB, Supplementary Table 8) revealed significantly elevated expression levels of IL-6 within IMs located in adjacent lung tissue to tumour tissue compared to IMs in distant areas of the lung within the same patients. Remarkably, these patients also exhibited ITGα5 expression at tumour sites. These data provide clinically relevant supportive evidence for our earlier findings regarding the significance of heightened IL-6 expression in IMs during tumour progression (Fig. 6a, b).

Collectively, these findings strongly indicate that ITGα5, packaged by specific cancer-derived EVPs, induces vascular permeability through the activation of IL-6 signaling and secretion from IMs. In turn, IL-6 compromises the integrity of the endothelial barrier, allowing for the extravasation of circulating cancer cells and metastasis formation in the lung (Fig. 6c).

## Discussion

Previous studies have established that the ability of EVPs to increase vascular permeability in the lung is primarily mediated through the direct effects of EVPs on endothelial cells. This in turn results in remodeling of endothelial tight junctions and adherent junctions and in the transfer of EVP microRNAs directly to endothelial cells^11,19,21,41,42^. Other studies have also shown that EVPs can transfer proteins to endothelial cells and that these proteins can then take part in alternating vascular permeability^22,23^.

Our present study unveils a novel mechanism in which the direct uptake of EVPs by endothelial cells does not have a discernible impact on vascular permeability. Instead, our findings point to an alternative, indirect mechanism whereby uptake of ITGα5-enriched tumour EVPs by IMs located close to the endothelium, is essential for vascular leakiness in the pre-metastatic lung. We show that, upon EVP uptake by IMs, EVPs stimulate the secretion of cytokines, notably IL-6, to foster endothelial permeability^38^. The depletion of IMs or blocking IL-6 *in vivo* significantly reduced the ability of EVPs to induce vascular leakiness and concurrently reduced the number of metastatic lesions in mice.

For this study, we employed an experimental metastasis model involving the intravenous injection of cells. While this model may not be a perfect indicator of metastatic events, it serves as a valuable tool for investigating the extravasation process within the lung and avoiding other EVP-independent effects of the primary tumour that have been shown to affect vascular leakiness. Moreover, it allows us to leverage the early onset of leakiness in the lung microenvironment, providing insights into the initial stages of metastatic cell infiltration and the factors influencing this crucial step in the metastatic cascade.

While B16F10, K7M2, and 4T1 tumours are all capable of metastasizing to the lung, the ability to induce vascular permeability varies widely among these different cancer models. Notably, in 4T1 tumours, despite high metastatic potential, the 4T1-derived EVPs induce relatively mild vascular permeability. Thus, the metastatic ability of 4T1 tumours likely relies on other mechanisms, such as the recruitment of neutrophils^43^. Nonetheless, the array of mechanisms that different tumour types exploit to successfully undergo the necessary steps to metastasize underscores the need to uncover more missing pieces of the metastasis puzzle. Indeed, even the mechanisms by which different tumours induce vascular permeability to enable extravasation and metastasis are not universal and can involve direct endothelial interactions, either by a variety of tumour-secreted factors or circulating tumour EVPs or, as our findings indicate, indirectly via EVP uptake and activation of immune mediators and subsequent disruption of endothelial barrier integrity11,17,19,21,41,42.

In our study, we identified a novel mechanism that implicates IMs as gatekeepers of metastatic progression. Interstitial and alveolar macrophages constitute the two primary populations of resident pulmonary macrophages. In contrast to AMs, which reside in close proximity to the epithelial alveolar cells within the alveoli, IMs are predominantly situated within the interstitial space that exists between the microvascular endothelium and alveolar epithelium^44,45^. This distinct localization underscores the potential influence that IMs may exert on the surrounding endothelial cells. Compared to alveolar macrophages, IMs are less frequent and relatively understudied, especially regarding their role in cancer. Prior mouse and human metastasis research suggested that IMs are capable of secreting pro-inflammatory cytokines, such as IL-6 and TNF-α, in both mice and humans^46–50^, consistent with our current study. With respect to cancer, several studies have demonstrated that IMs represent a significant proportion of tumourassociated macrophages (TAMs) within pulmonary tumours and that their presence has been closely correlated with tumour cell growth *in vivo*^51^. However, our findings demonstrating the role of IMs in inducing vascular leakiness and subsequently promoting extravasation and metastasis are novel.

Importantly, we identified ITGα5 as the EVP cargo necessary and sufficient to induce vascular permeability and facilitate metastasis. ITGα5 loss in cells and EVPs reduces their ability to increase vascular leakiness, cancer cell extravasation, and metastasis to the lung. The absence of ITGα5 on EVPs does not impair the ability of IMs to uptake EVPs but significantly hinders activation of STAT signaling and IL-6 secretion, which consequently diminishes their effect on adjacent vasculature.

ITGα5 is known to recognize and bind to the RGD sequence (Arg-Gly-Asp), which serves as a key mediator of cell adhesion on fibronectin- and osteopontin-binding receptors. This interaction has been implicated in the regulation of differentiation across diverse cell types^52–54^. However, if and how ITGα5 impacts these established pathways in lung IMs remains to be explored.

It is well established that IL-6 enhances endothelial permeability in *in vitro* models,^55^, primarily by altering VE-cadherin, and tight junction proteins^56^. Our findings that vascular permeability is induced via secretion of IL-6 from IMs, and that blocking IL-6 results in a notable reduction in both vascular leakiness and metastasis *in vivo*, not only support established *in vitro* findings but also suggest a mechanistic basis for how tumour EVPs can prime and exploit cell populations within the pre-metastatic microenvironment to facilitate metastasis.

Not surprisingly, ITGα5 is upregulated in a spectrum of tumours and is closely associated with unfavorable prognostic outcomes, including lung cancer^57,58^. ITGα5 has been demonstrated to play a pivotal role in driving tumour progression and metastasis^57,59–62^. Additionally, ITGα5 has also been linked to the promotion of angiogenesis and exhibits a correlation with heightened immune infiltration within the tumour microenvironment^63,64^. Although the precise role of ITGα5 in influencing vascular permeability remains unexplored, some evidence suggests an increase in the levels of pro-inflammatory cytokines, IL-6 and TNF-α, in tumours that express elevated levels of ITGα5^64,65^. We confirmed that this elevation of pro-inflammatory cytokines is associated with an observed increase in permeability in our *in vitro* models.

Furthermore, in COVID-19 patients, the EC inflammatory phenotype and permeability depend on ITGα5. Consistent with our findings, inhibition of ITGα5 decreased EC permeability and IL-6 secretion in COVID-19 patients^66,67^. Additionally, the SARS-CoV-2 virus infects and activates interstitial macrophages IMs, leading to a cytokine storm, including IL-6 secretion^68^. Collectively, these findings suggest that our observations indicating that KO of ITGα5 in EVP reduces IM IL-6 levels and subsequently diminishes vascular permeability and the establishment of metastatic lesions, may have common implications in other diseases such as COVID-19.

Our research also provides the first detailed study of the kinetics of vascular permeability induction by both tumours and their EVPs, and reveals a remarkably short time frame required for the induction of vascular permeability in the lung. This notion has provided us with an opportunity to delve into the specific influence of EVPs (resulted from a single injection) on the permeability of lung endothelial cells and the extravasation of cells while minimizing the confounding effects associated with EVP treatment. This is in contrast to previous studies, which have often demonstrated the importance of vascular leakiness following tumour growth or multiple injections of EVPs *in vivo*^11,21^, which could introduce other variables such as immune infiltration and modulation, angiogenesis, and ECM remodeling^13^. Our research has highlighted the potent impact of a single EVP injection, emphasizing the critical role of vascular leakiness in the processes of extravasation and metastasis.

In summary, our study offers a novel perspective regarding the influence of IMs in mediating the effects of tumour-derived EVPs on both vascular permeability and the progression of metastatic events. We identify new interactions between EVPs, IMs, IL-6, and ITGα5, elucidating their significant roles in the regulation of vascular permeability and the advancement of metastatic processes, thus presenting several potential therapeutic opportunities.

## Materials and methods

### Cell lines and cell culture

B16F10, K7M2 and 4T1 cells were purchased from American Type Culture Collection (ATCC), These cells were cultured in DMEM (Corning) supplemented with 10% FBS (Gibco) and 1× penicillin/streptomycin (100 U ml−1 of penicillin and 100 μg ml−1 of streptomycin, Thermo Fisher Scientific). The 67NR cell line was obtained from F. Miller, and cultured in RPMI (Corning) supplemented with 10% FBS and 1× penicillin/streptomycin. The mouse melanocyte Melan-A line was obtained from The Wellcome Trust Functional Genomics Cell Bank and cultured in RPMI supplemented with 10% FBS, 1× penicillin/streptomycin and 0.2 μM 12-Otetradecanoylphorbol-13-acetate (TPA) (Sigma). Human pulmonary artery endothelial cells (HPAEC) were obtained from PromoCell and were cultures in Endothelial Cell Growth Medium 2 (PromoCell). All studies were done on cells between passages 3–8. Primary osteoblasts were isolated from mouse bones, and primary interstitial macrophages were isolated from mouse lung as described below. When collecting conditioned media for EVP isolation, FBS was first depleted of EVPs by ultracentrifugation at 100,000g for 4 h. Cells were cultured in EVP-depleted media for 3 days and supernatant was collected for EVP isolation. Cells were maintained in a humidified 37°C incubator with 5% CO2, and cell lines routinely tested and confirmed to be negative for mycoplasma.

### EVP isolation and characterization

EVPs were purified by sequential ultracentrifugation; cell contamination was removed from 3–4 days cell culture supernatant or resected tissue culture supernatant by centrifugation at 500 × g for 10 min. To remove apoptotic bodies and large cell debris, the supernatants were then spun at 3,000 × g for 20 min, followed by centrifugation at 12,000 × g for 20 min to remove large microvesicles. Finally, EVPs were collected by ultracentrifugation in 38 or 94 mL ultracentrifugation tubes (#355631 or #355628 Beckman Coulter) at 100,000 × g for 70min. EVPs were washed in PBS and pelleted again by 100,000 × g ultracentrifugation in 70Ti or 45Ti fixed-angle rotors in a Beckman Coulter Optima XE or XPE ultracentrifuge at 10 μC. The final EVPs pellet was resuspended in PBS, and protein concentration was measured by BCA (Pierce, Thermo Fisher Scientific).

### EVPs labeling and biodistribution assessment

EVPs were labelled with the near-infrared dye CellVue^™^ Burgundy (eBioscience) or PKH67 (Sigma) following the manufacturer’s protocol, followed by washing with 20 ml of PBS and pelleting by ultracentrifugation at 100,000g for 70 min at 10 °C. Labelled nanovesicles (10 μg) resuspended in 100 μl of PBS, or an equivalent volume of mock reaction mixture, were retro-orbitally injected into naive mice. At 1 h post-injection, tissues were collected and analyzed using flow cytometry or Immunofluorescence. All animal experiments were performed in compliance with ethical regulations and in accordance with WCM institutional, IACUC and AAALAS guidelines, approved for animal protocol 0709–666A.

### Immunofluorescence staining for tissues and cells culture.

For Tissues - Two lung lobes from each mouse was fixed in a 4% PFA in PBS overnight, then transferred to a 30% sucrose in PBS solution for an additional overnight. The next day the tissue were incubated for 1h in 1:1 30% sucrose Tissue-tek O.C.T. embedding compound, followed by embedding Tissue-tek O.C.T. embedding compound. Blocks were frozen on a dry-ice bath. For immunofluorescence, 10 μm O.C.T tissue cryosections were stained by standard immunofluorescence protocol. Briefly slides were dried and cryosections blocked with blocking solution (PBS containing 3% BSA and 0.2% Triton X-100), and then incubated with primary antibodies (**Supplementary Table 9**) overnight at 4 °C. Slides were then washed with PBS and incubated with secondary antibodies (**Supplementary Table 9**) for 1h, stained with DAPI (1 μg/ml), and mounted with ProLong Diamond Antifade Mountant (Thermo Fisher Scientific, P36970).

For HAPEC cells – Cells were fixed with 4% PFA, followed by permeabilization with 0.2% triton for 2 minutes. Blocked and stained as described above. Slides were visualized by LSM 880 Laser Scanning Confocal Microscope (Zeiss), with 40x DIC objective. Images were viewed and analyzed with Zen Blue (Zeiss).

For human sections - Autofluorescence was quenched using Quenching Kit (Vector Laboratories^™^,SP-8400–15).

Intensity of each cell was measured and divided by the cell area to get the mean intensity per cell. Mean intensities were compared between adjacent and distant sections of the same patient.

### histological analysis

For histological analysis of lung with H&E staining, lung tissues were fixed in 4% PFA overnight at 4 °C and subjected to paraffin embedding. Paraffin-embedded lung tissues were sectioned at 7-μm thickness, and sections were processed for H&E staining and mounted with VectaMount medium (Vector Laboratories). Slides were scanned by PANNORAMIC 250 Flash (V2.6, 3DHISTECH ltd) with a 20x/0.8NA (Zeiss). The number of metastases was counted manually, each lesion’s area was measured by FIJI software^69^.

### Flow cytometry

Digestion and staining followed a previously published protocol^70^. Briefly, Lungs tissues were minced and then digested at 37 °C for 1 hour with an enzyme cocktail: collagenase A (1mg/ml), dispase (1mg/ml) and DNaseI (0.1mg/ml) (Roche Sigma- Aldrich). Single-cell suspensions were filtered and washed with PBS containing 2 mM EDTA and 3% BSA. isolated cells were subjected to red blood lysis and incubated with the primary antibodies described in **Supplementary Table 9**. To define cell viability, DAPI (Thermo Fisher) was used. Data were acquired by Aurora (Cytek) and analyzed by FCS express 7 research (Denovo software).

### Transmission electron microscopy (TEM)

For EVPs negative staining TEM analysis, 0.1 mg/ml of EVPs in PBS were placed on a formvar/carbon coated grid and allowed to settle for 1 min. The sample was blotted and negatively stained with 4 successive drops of 1.5% (aqu) uranyl acetate, blotting between each drop. Following the last drop of stain, the grid was blotted and air-dried. Grids were imaged with a JEOL JSM 1400 (JEOL, USA, Ltd, Peabody, MA) transmission electron microscope operating at 100Kv. on a Veleta 2K × 2K CCD camera (Olympus-SIS, Munich, Germany).

### Interstitial macrophages isolation

Lungs were digested and stained as described above. IMs were stained with the listed Abs (**Supplementary Table 9**) and sorted by BD FACS Melody, typically 100–300,000 cells were collected from each Lung. Cells were cultured with RPMI with 10% FBS, GM-CSF (20ng/ml), 1 mM Sodium Pyruvate, and 1x penicillin/streptomycin for 24h, and subjected to EVPs treatment.

### Primary osteoblast isolation and culture

Primary mouse osteoblasts were isolated from BALB/c mouse bones as previously described^71^. In brief, 7-week-old female mice were euthanized and bone tissues including the tibia, femur and humerus were collected. Bone marrow was removed by flushing with basal medium αMEM (BioConcept) containing 2.2 g of NaHCO3, 1× penicillin/streptomycin, 2 mM of L-glutamine, 0.375× MEM amino acids (BioConcept) and 10% EVP-depleted FBS). Bone tissues were then cut into small pieces and seeded into a 10-mm dish with 10 ml of digestion medium (basal medium containing 1 mg/ml of collagenase II (Sigma, C6885)). After incubation at 37 °C for 90 min, the digestion medium was replaced by basal medium to allow the cells to migrate from bone pieces and attach to the dish. Three days later, cells were detached with collagenase I (Thermo Fisher Scientific) solution followed by TrypLE Express Enzyme (Thermo Fisher Scientific). When cells reached passage 3 to 5, the isolation of osteoblasts was confirmed by measuring of mineralization using Alizarin Red-S staining, and >97% of cultured cells were osteoblasts (data not shown). The supernatant from passage 3 to 5 osteoblasts was collected for EVP isolation.

### RNA extraction and RT–qPCR analysis

Total RNA was purified using TRIzol (Thermo Fisher Scientific) according to the manufacturer’s instructions, and quantified by OD260 nm/OD280 nm measurement. For RNAseq, total RNA was extracted using TRIzol reagent and RNA was further purified using RNeasy Mini kit including a DNase digest following the manufacturer’s instructions (Qiagen).

For RT–qPCR analysis, 100–500 ng of total RNA was used for cDNA synthesis using the highcapacity cDNA reverse transcription kit with RNase inhibitor (Thermo Fisher Scientific) following the manufacturer’s instructions. Ten nanograms of cDNA were used for RT–qPCR reactions using SYBR Green (Thermo Fisher Scientific) and gene-specific primers. GAPDH was used as an internal control. RT–qPCR was performed on a CFX384 Touch Real-Time PCR System (Bio-Rad). Primers used in RT–qPCR analysis are listed in **Supplementary Table 10**.

### Cytokine array

Interstitial macrophages were isolated from mouse lung and cultured overnight. Cells were treated with EVP`s (1μg/ml) for 3h. The conditioned media were then collected for cytokine array analysis. Cytokine array analysis was carried out using the Proteome Profiler Mouse Cytokine Array Kit, Panel A (R&D) according to manufacturer’s instructions. The blot was analyzed by ChemiDoc^™^ XRS+ (Bio-Rad), and the pixel densities on the developed X-ray film was quantified using Fiji.

### Western blot analysis

Total cell lysate (TCL) and EVPs lysate were generated by lysing cell in RIPA buffer (Sigma Aldrich). Lysates were cleared by centrifugation at 12,000g for 15 min, 4°C. The clear lysate was mixed with SDS sample buffer. The samples (both TCL and EVPs) were boiled for 5 min. 2.5–10 μg of input were separated on a Novex 4%−12% or 4%−20% Bis-Tris Plus Gel (Life Technologies) and transferred onto a nitrocellulose membrane (0.45 μm, Bio-Rad). Membranes were blocked for 1 hour at RT followed by primary antibody incubation overnight at 4°C, and secondary antibody for 1 hour at RT. The blot was analyzed by ChemiDoc^™^ XRS+ (Bio-Rad), and analyzed by Image Lab (V6.1, Bio-Rad). All used antibodies and dilution are listed (**Supplementary Table 10**).

### *In vivo* models:

All mouse work was performed in accordance with institutional, IACUC and AAALAS guidelines, by the animal protocol 0709–666A. All animals were monitored for abnormal tissue growth or ill effects according to AAALAS guidelines and euthanized if excessive deterioration of animal health was observed. Both BALB/cJ and C57BL/6 mice were obtained from the Jackson Laboratory. All mice were bred and housed in the Biological Resource Centre animal facility under Specific Pathogen-Free conditions. All mice used in the *in vivo* experiments were aged 7 – 12 weeks. No statistical method was used to pre-determine sample size. No method of randomization was used to allocate animals to experimental groups. The investigators were not blinded to allocation during experiments and outcome assessment. Mice that died before the predetermined end of the experiment were excluded from the analysis.

BALB/cJ mice aged 6–8 weeks were used for the implantation of mouse breast cancer cell lines (4T1) and mouse osteosarcoma cell line (K7M2), as well as treatment with osteoblast-EVPs, 4T1 and K7M2 EVPs; C57BL/6 mice aged 6–8 weeks were used for the implantation of mouse melanoma cell lines B16F10, and treatment with B16F10 and Melan-a EVP`s. No statistical method was used to pre-determine the sample size and no method of randomization was used to allocate animals to experimental groups.

For tumor cell implantation, 5 × 10^5^ of 67NR or 4T1 cells in 50 μl of PBS were injected into the mammary fat pad of BALB/c mice; 5 × 10^5^ of B16F10 cells in 100 μl of PBS were subcutaneously injected into C57BL/6 mice; 1 × 10^6^ of K7M2 cells in 10 μl of PBS were injected into the tibias of BALB/c mice. Mice were euthanized 2 weeks after tumor cell implantation for tissue collection. Mice injected with an equivalent volume of PBS following the same procedure were used as the control group.

For education experiment – 10 μg of control KO or ITGα5 KO EVP every other day for 3 weeks. After 3 weeks 1 × 10^6^ of K7M2 cells in 10 μl of PBS were injected into the tibias of BALB/c mice. Mice were euthanized 4 weeks after tumor cell implantation for tissue collection.For experimental lung metastasis model, 5×10^4^ B16F10 and 4T1 tumor cells were tail vein injected, and lung metastatic foci were measured 14 days later. For the K7M2, 5 ×10^5^ were tail vein injected and mice were euthanized after 2.5 weeks. When described, cells injected 1h following EVP`s administration.

For Alveolar macrophages (AM) depletion in naive mice, liposome or clodronate (Liposoma) was intra-nasally injected into mice at a dosage of 60 μl per mouse. At 72 h post injection, mice were euthanized to confirm the efficiency of AM depletion, or injected with EVP treatment to assess leakiness.

For *in vivo* neutralization assays, InVivo anti-CSF1R (BE0213,) or InVivo anti LY6G (BE0320) or InVivo anti IL-6 (BE0046) or a or InVivo anti-TNFa (BE0058) or InVivo anti IgG Isotype control (BE0090) from BioXCell were intraperitoneally injected into the mice at a dosage of 200 μg per mouse 18h before EVP`s treatment. Anti-CXCL2 (MAB452), CCL3 (AB-450-NA) were purchased from R&D were intraperitoneally injected into the mice at a dosage of 50μg per mouse 18h before EVP`s treatment.

For *in vivo* conditioned medium education, EVP-depleted conditioned medium obtained after EVP isolation (that is, the media supernatant after first spin of 100,000g for 70 min as described above) from cultured cancer cells was concentrated using Amicon Ultra-15 centrifugal filters with 10 kDa cutoff (Millipore, UFC901024) at 4,000g at 4 °C to a volume of 100 μl and used for each injection.

### *In vitro* permeability assay

10–20*10^5 HPAEC cells was seeded on the top well of Rat tail collagen treated (100 μg/mL) transwell filters (3.0-μm pore size; VWR). The cells grown for 3–5 days until reach confluent. The cell treated with EVPs or secretome of IM with Dextran 25 μg/mL (Rhodamine B, 70,000 MW, Lysine Fixable, Thermo scientific) for 60 min. Following 60 minutes, the medium in the bottom well was collected the appearance of fluorescence was measured at excitation 555/emission 580 wavelength.

### *In vivo* permeability assay

10 μg of purified EVPs in a volume of 100 μl, was injected retro-orbitally to anaesthetized 7–8 week-old mice. One hour after EVP`s treatment, mice were injected with 0.5 mg of Dextran (Rhodamine B, 70,000 MW, Lysine Fixable, Thermo scientific) retro-orbitally. One hour after dextran injection, mice were euthanized and perfused with of PBS to remove excess dye. Lungs were dissected and fixed in a mix of 4% PFA overnight for further processing.

### Recombinant IL-6

Recombinant IL-6 purchased from Peprotech and used in the concertation on 40nM for 1 hour for the *in vitro* assays. For *in vivo* assay 500nM IL-6 injected Intraperitonially 1 hour before dextran administration.

### *Ex vivo* whole lung imaging assay

B16f10 cells stained with CellTracker^™^ Green CMFDA Dye (Invitrogen, catalog number: C2925) according to the manufacturer’s instructions. Labeled B16F10 cells (5 × 10^5^) were injected intravenously into tail veins of mice 1 hour following EVPs/PBS injection. After 4 days, isolated lungs were placed in a specially designed chamber with a coverslip glass (0.16–0.19 mm thick) at its bottom. To visualize lung endothelium, anti-CD31–PE antibody (50 mg/kg; 102408, BioLegend) was injected in retro-orbitally 5 minutes before sacrifice. Lungs were inflated with 0.5 ml of air and remained inflated during the imaging^72–74^. Tumor cell extravasation was evaluated visually from microscopic FOV or through reconstruction of tumor cells and vessel surface with Imaris software.

### Generation of ITGa5 and NCAM1 knockout in B16F10 and K7M2 cell lines

Knockout in B16F10 and K7M2 cells was achieved by infecting cells using lentivirus (lentiCas9-Blast Addgene, #52962) for creating Cas9 expressing stable cell lines. Following 2 weeks we used lentivirus (lentiGuide-Puro Addgene, #52963) carrying guide RNA targeting mouse integrin (sequences can be found in the **Supplementary Table 9**. The single guide RNA targeting sequence was chosen using CHOPCHOP sgRNA Designer (https://chopchop.cbu.uib.no/). As a control, B16F10 cells were infected with lentiCRISPR empty-vector virus.

Lentivirus was produced by co-transfection of the lentiviral expression vector and viral packaging/envelope plasmids, including pMDLg/pRRE (Addgene, #12251), pMD.2G (Addgene, #12259) and pRSV-Rev (Addgene, #12253) into 293T cells using TransIT-X2^®^ Transfection Reagent (Mirus).

Knockdown of Cdh2 was achieved by transfected the cell with ON-TARGETplus Mouse CDH2 siRNA (Horizon, cat: L-040206-00-0005) with TransIT-X2^®^ Transfection Reagent (Mirus). Condition media was collected 48h after transfection. ON-TARGETplus Non-targeting Control Pool siRNA (Horizon, cat: D-001810-10-05) was used as control.

### Human Studies

Fresh human tissues (tumor, lung adjacent to the tumor, lung distant from the tumor) were harvested from patients with lung cancer who underwent surgery at Memorial Sloan Kettering Cancer Center (MSK) and collected through the MSK Biobank, Department of Pathology. The tissues were fixed in 4% paraformaldehyde at 4°C overnight, then cryoprotected and embedded in OCT. All patients provided written informed consent to tissue donation according to the protocols approved by the Institutional Review Board of MSK (IRB 12–245).

### Mass-Spectrometry

Enriched EVPs sample (5 μg) was dried by vacuum centrifugation and re-dissolved in 30–50 uL of 8M Urea/50mM ammonium bicarbonate/10 mm DTT. Following lysis and reduction, proteins were alkylated using 20 or 30 mM iodoacetamide (Sigma). Proteins were digested with Endopeptidase Lys C (Wako) in < 4 M urea followed by trypsination (Promega) in < 2 M Urea. Peptides were desalted and concentrated using Empore C18-based solid phase extraction prior to analysis by high resolution/high mass accuracy reversed phase (C18) nano-LC-MS/MS. Typically, 30% of samples were injected. Peptides were separated on a C18 column (12 cm / 75 mm, 3 mm beads, Nikkyo Technologies) at 200 or 300 nl/min with a gradient increasing from 1% Buffer B/9 5% buffer A to 40% buffer B/60% Buffer A in typically 90 min or 120 min (buffer A: 0.1% formic acid, buffer B: 0.1% formic acid in 80% acetonitrile). Mass spectrometers (Q-Exactive, Q-Exactive Plus, Q-Exactive-HF or Fusion Lumos, Thermo Scientific) were operated in data dependent (DDA) positive ion mode.

### RNA sequencing

For *in vivo* RNA-seq - Lungs were digested and stained as described above. IM were stained with the listed Abs (**Supplementary Table 10**) and sorted. by BD FACSMelody, typically 10–40,000 cells were collected from each lung. For *in vitro* RNA-seq – sorted IM were treated with EVPs for 3 hours before adding Trizol. Total RNA was isolated using Trizol and the RNeasy mini kit (Qiagen, Hilden, Germany). Following RNA isolation, total RNA integrity is checked using a 2100 Bioanalyzer (Agilent Technologies, Santa Clara, CA). RNA concentrations are measured using the NanoDrop system (Thermo Fisher Scientific, Inc., Waltham, MA). Preparation of RNA sample library and RNA-seq were performed by the Genomics Core Laboratory at Weill Cornell Medicine. Large quantity of sample is accomplished by Agilent high throughput sample preparation Bravo B system automated with Illumina Stranded mRNA Sample Library Preparation kit (Illumina, San Diego, CA, PN 20040534), according to the manufacturer’s instructions. The normalized cDNA libraries are pooled and sequenced on Illumina NextSeq 2000 sequencer with P2 Kit at pair-end 50 cycles. The raw sequencing reads in BCL format are processed through bcl2fastq 2.19 (Illumina) for FASTQ conversion and demultiplexing.

### Bioinformatical analysis

High resolution/high mass accuracy nano-LC-MS/MS data are processed using Proteome Discoverer 1.4.1.14/Mascot 2.5 software. The relative abundance of a given protein is calculated from the average area of the three most intense peptide signals. For the proteins identified by multiple UniProt ID, the probe (based on UniProt ID) values are collapsed at the protein level using the probe with the maximum intensity. Quantile normalization can be considered if observed changes across samples are due to unwanted technical variability. The proteomic expression data are processed using the ‘Limma’ package of the open-source R program (https://www.r-project.org). Proteomic expression data are imported and are normalized using the ‘normalizeBetweenArrays’ function (method=quantile). Heatmaps and clustering are frequently used for data visualization. A heatmap is generated using the GENE-E software (https://software.broadinstitute.org/morpheus/). pathway analysis was performed with https://metascape.org/^75^.

Gene Set Enrichment Analysis (GSEA) is used to identify significant biological functions or pathways related to identified proteins. Briefly, GSEA ranks all proteins according to their differential expression levels by signal-to-noise statistic, (μA - μB)/(αA + αB) where μ and α represent the mean and standard deviation of proteomic expression, respectively, for each class. Next, GSEA calculates the Kolmogorov-Smirnov statistic to evaluate whether proteins from a pre-determined pathway are significantly overrepresented towards the top or bottom of the ranked gene list. Gene sets from the Molecular signatures database (MSigDB, http://www.broadinstitute.org/gsea/msigdb/) were used for GSEA (H: hallmark gene sets; C2:KEGG: canonical pathways from Kyoto Encyclopedia of Genes and Genomes [KEGG] pathway database; C5: gene sets based on Gene Ontology [GO] term). All statistical tests were performed using the Graphpad Prism (v9.4.1).

### Data availability

RNA-seq raw data and associated processed data files that support the findings of this study have been deposited in the Gene Expression Omnibus under accession codes GSE261139 (https://www.ncbi.nlm.nih.gov/geo/query/acc.cgi?acc=GSE261139)

### Statistical analysis

All statistical analysis was performed with GraphPad -Prism 9 software. An appropriate statistical test (student t-test or 2-way ANOVA) was used to determines statistical significance (*p < 0.05 **p <0.01 ***p <0.001 ****p <0.0001).

## Extended Data

**Extended Fig 1:** **a**. Representative immune-histochemistry imaging of mouse lung exhibit expression of vWF (top) and VE-cadherin (bottom). Scale bar: 1000 μm. **b**. Representative images of *in vivo* vascular permeability. DAPI-stained nuclei appear in blue (×20 magnification). Data represent the mean ± SEM. (*n* = 3). Scale bar: 100 μm. **c**. FACS analysis of the uptake of EVPs from different cancer cell lines in mouse lung, 1h after EVPs injection. Data represent the mean ± SEM. (*n* = 3), p<0.01. **d**. Representative images (left) and associated statistical analysis (right) of *in vivo* vascular permeability determined by the appearance of intravenously injected dextran (red). DAPI-stained nuclei appear in blue (×20 magnification). (*n* = 3), p<0.01. Scale bar:100 μm.

**Extended Figure 2:** **a**. Representative images of *in vivo* vascular permeability determined by the appearance of intravenously injected dextran (red). DAPI-stained nuclei appear in blue (×20 magnification). (*n* = 2), p<0.01. Scale bar:100 μm. **b**. Representative images of mice’s lungs at two weeks after tail vein injection with 50,000 B16F10 cells (left) and associated statistical (right) of relative number of macro-mets compared to PBS. Data represent the mean ± SEM (*n* = 1), p<0.05. **c**. Representative immunofluorescence images of fresh whole lung. The arrow shows the labeled cells in the lung. Scale bar: 500 μm.

**Extended Figure 3:** **a**. Representative image of FACS analysis showing the percentage of EVPs+ cells relative to the total cell count. **b**. Gating strategy of FACS experiment. **c**. Representative images (left) and associated statistical analysis (right) of immune cell population in the lung following depletion of alveolar macrophages (top), interstitial macrophages (middle), and neutrophils (bottom). **d**. Representative images of *in vivo* vascular permeability determined by the appearance of intravenously injected dextran (red). DAPI-stained nuclei appear in blue (×20 magnification). Data represent the mean ± SEM. (*n* = 2). p<0.01. Scale bar: 100 μm.

**Extended Figure 4:** **a**. Effect of 4T1 and K7M2 EVPs on the permeability of the HPAEC monolayers by *in vitro* permeability assay. Data represent the mean ± SEM. (*n* = 2). p<0.01. **b**. GESA analysis for the pathways enriched in Con Vs B16F10. **c**. Cytokine array of the secretome from IMs 3h after treatment with B16F10/K7M2/4T-1 EVPs or PBS. **d**. RT-PCR analysis from IMs after treatment with PBS, 4T1, melan-A, K7M2, and B16F10 EVPs. Data represent the mean ± SEM. (*n* = 2). **e-f**. Representative images (left) and associated statistical analysis (right) of *in vivo* vascular permeability determined by the appearance of intravenously injected dextran (red). DAPI-stained nuclei appear in blue. (×20 magnification). Data represent the mean ± SEM. (*n* = 2), p<0.01. Scale bar: 100 μm.

**Extended Figure 5:** **a**. Heatmaps of adhesion molecules expression in EVPs from different cancer cell lines. **b**. Western blot analysis of CHD2 and NCAM1. CD9 was used as a loading control. **c**. Western blot analysis of CHD2 and NCAM1 after knockdown/knockout. CD9 was used as a loading control. **d**. EVPs` images by transition electron microscopy. **e**. Representative images of *in vivo* vascular permeability determined by the appearance of intravenously injected dextran (red). DAPI-stained nuclei appear in blue (×20 magnification). (*n* = 2). Scale bar: 100 μm. **f**. Nanosight tracking analysis (NTA), the histogram is an average of 3 readings (top). EVPs` size and concertation as measured by NTA. Data represent the mean ± SEM. (*n* = 3), p<0.05. **g**. FACS analysis showing the percentages of EVPs+ cells from total live cells. Top: B16F10 EVPs. bottom: K7M2 EVPs. Data represent the mean ± SEM. (*n* = 3). **h**. FACS analysis showing the percentages of CD31+ and CD45+ cells from EVP+ cells. Top: B16F10 EVPs. bottom: K7M2 EVPs. Data represent the mean ± SEM. (*n* = 2). **i**. FACS analysis showing the percentages of IM+ cells from F4/80+ cells. Top: B16F10 EVPs. bottom: K7M2 EVPs. Data represent the mean ± SEM. (*n* = 2). **j**. Analysis of tumour weight of B16F10 tumours (ITGα5 KO Vs CON KO) at two weeks after tumour implantation. Data represent the mean ± SEM. (*n* = 2). **k**. Principal component analysis (PCA) of gene expression in sorted IMs treated with PBS, CON KO, or ITGα5 KO. (*n* = 2). **l**. GESA analysis for the pathways enriched in ITGα5 KO Vs CON KO.

## Data Availability

MS data and RNAseq data can be found in extended data. All other data supporting the findings of this study are available from the corresponding authors on reasonable request.
